# Dysregulated phosphoinositide 3-kinase signaling in microglia: shaping chronic neuroinflammation

**DOI:** 10.1186/s12974-021-02325-6

**Published:** 2021-11-27

**Authors:** Erskine Chu, Richelle Mychasiuk, Margaret L. Hibbs, Bridgette D. Semple

**Affiliations:** 1 Department of Immunology and Pathology, Central Clinical School, Monash University, Level 6, 89 Commercial Road, Melbourne, VIC 3004 Australia; 2 Present Address: Department of Neuroscience, Central Clinical School, Monash University, Level 6, 99 Commercial Road, Melbourne, VIC 3004 Australia; 3 Department of Neurology, Alfred Health, Prahran, VIC 3181 Australia; 4 Department of Medicine (Royal Melbourne Hospital), The University of Melbourne, Parkville, VIC 3050 Australia

**Keywords:** Neurodegenerative diseases, PI3K, AKT, Cell signaling, Innate immune system, Glia

## Abstract

Microglia are integral mediators of innate immunity within the mammalian central nervous system. Typical microglial responses are transient, intending to restore homeostasis by orchestrating the removal of pathogens and debris and the regeneration of damaged neurons. However, prolonged and persistent microglial activation can drive chronic neuroinflammation and is associated with neurodegenerative disease. Recent evidence has revealed that abnormalities in microglial signaling pathways involving phosphatidylinositol 3-kinase (PI3K) and protein kinase B (AKT) may contribute to altered microglial activity and exacerbated neuroimmune responses. In this scoping review, the known and suspected roles of PI3K-AKT signaling in microglia, both during health and pathological states, will be examined, and the key microglial receptors that induce PI3K-AKT signaling in microglia will be described. Since aberrant signaling is correlated with neurodegenerative disease onset, the relationship between maladapted PI3K-AKT signaling and the development of neurodegenerative disease will also be explored. Finally, studies in which microglial PI3K-AKT signaling has been modulated will be highlighted, as this may prove to be a promising therapeutic approach for the future treatment of a range of neuroinflammatory conditions.

## Background

Defined as an inflammatory response within the central nervous system (CNS), “neuroinflammation” refers to activation of the brain’s innate immune system following a disturbance to homeostasis, such as injury, infection, or the onset of neurodegeneration, which frequently occurs with aging. This inflammatory response represents a highly sophisticated biological program comprised of both pro- and anti-inflammatory arms. The pro-inflammatory response, driven by the release and subsequent signaling of multiple soluble factors known as cytokines, triggers cell activation, promotes vascular permeability and induces leukocyte recruitment, accumulation, and activation, whereas the anti-inflammatory response initiates wound healing and regenerative processes for recovery [1]. In the brain, the neuroinflammatory response is controlled by various glial cells including microglia, astrocytes, and oligodendrocytes [2]. Constituting around 7% of the entire glial population in humans, microglia are recognized as key orchestrators of neuroinflammation, and are capable of participating in both pro- and anti-inflammatory responses [3–5].

The purpose of the neuroinflammatory response is to re-establish homeostasis, and it is typically self-limiting. However, when this response is not properly inactivated, ongoing neuroinflammation can result in deterioration of healthy tissue, which may severely impair physiological functioning. Aberrant microglial responses and persistent neuroinflammation have been implicated in neurodegenerative diseases, such as Alzheimer’s disease and Parkinson’s disease, as well as psychological disorders [6, 7]. In addition, following severe and repeated traumatic brain injuries (TBI), prolonged neuroinflammation is thought to limit neuronal repair and promote secondary neurodegeneration [8–10]. The cellular and biomolecular cascades that induce excessive neuroinflammation are complex and not yet fully understood; however, recent studies have suggested that unchecked microglial activation can lead to the development of chronic neuroinflammation [11–13]. In fact, it is hypothesized that the contribution of microglia in shifting the homeostatic neurological microenvironment to an inflammatory one likely exceeds that of other glial cells; microglia generate rapid responses to subtle changes in the CNS, and abnormal microglial responses are consistently correlated with neurological disorders [14–16].

The PI3K-AKT cascade is a highly conserved intracellular signaling pathway present in all eukaryotic cells, acting as a central node for transducing extracellular stimuli that involves phospholipid and protein phosphorylation of various downstream substrates to induce changes in cellular responses. This pathway orchestrates growth, motility, survival, and metabolism, as well as coordinating defense mechanisms in the immune system [17–20]. In the brain, AKT activity is also crucial for neuronal development and glial cell activities, with aberrant PI3K-AKT signaling implicated in various conditions including cancer, neurodegeneration, and neuroinflammatory diseases [21].

In view of the central role of microglia in establishing chronic neuroinflammation and neuropathology related to disease development, this review aims to summarize the current understanding of how PI3K-AKT signaling facilitates the microglial response, and how aberrant activity in this pathway may promote chronic neuroinflammation. We also consider how manipulation of AKT signaling can induce beneficial, anti-inflammatory responses to counteract the development of neuroinflammation; and highlight knowledge gaps as well as future directions of research*.*

## Microglia as regulators of the neuroinflammatory response

Microglia are an integral component of the neuroimmune system and act as one of the first lines of immune defense. They are described as the “macrophages of the brain” due to their similarity in function and biomarker expression, to monocyte-derived macrophages [22]. Under normal conditions, microglia are a heterogeneous population with differences in their functional signatures, phenotype, and population density across adult brain regions, which change with age [23–28]. Within a homeostatic context, microglia exhibit a “resting” ramified phenotype, whereby they continuously survey their microenvironment by extending and retracting their processes [29]. Upon detecting pathological insults or disturbances to homeostasis, resting microglia transition to an “activated” phenotype by undergoing morphological and biological changes [29, 30]. Activated microglia are characterized by a swollen ameboid shape with an enlarged cell body and truncated processes, as well as increased surface expression of ionized calcium binding adaptor molecule 1 [30]. More recently, single cell-based RNA sequencing on human and mouse microglia revealed a distinct gene signature in activated microglia within the context of neurodegenerative diseases and head injuries [25, 28, 31, 32]. In addition, they demonstrate a highly malleable, reactive inflammatory response [33]. While historically this response was classified as either pro- or anti-inflammatory, it is increasingly evident that activated microglia can simultaneously mediate both types of responses and can readily switch between phenotypes based on the nature of the stimuli [34].

The concept of “M1” and “M2” polarization was initially coined to describe the ability of macrophages to alter their phenotype in vitro in response to cytokines, such as interferon-γ or interleukin (IL)-4, becoming classically activated or alternatively activated, respectively. However, more recent in vivo analyses of both macrophages and microglia in experimental rodents has revealed this distinct description no longer holds up; their responses are more heterogeneous and occur in a continuum between pro- and anti-inflammatory states, such as concurrent expression of both inflammatory markers during acute responses to TBI and lipopolysaccharide (LPS) [4, 35–37].

Typically, microglial activation occurs in a transient manner that is intended to protect the CNS and aid recovery, by initiating both components of the inflammatory response in a temporal and spatially restricted manner. However, sustained or uncontrolled activation can have deleterious consequences. Chronic activation of microglia can result in robust changes to their inflammatory profile, associated with the continual release of neurotoxic inflammatory mediators, such as inducible nitric oxide synthase (iNOS) and pro-inflammatory cytokines, including, IL-1 and tumor necrosis factor (TNF)-α [38–40]. Various experimental studies of neurodegenerative diseases have identified populations of activated, pro-inflammatory microglia, whose presence is associated with poorer prognosis and worsened pathology [41–45]. Furthermore, clinical studies using magnetic resonance imaging revealed persistent microglial activation in patients several years after head injuries; with chronic microglial activation being associated with increased neuroinflammation and exacerbated cognitive impairments [46]. A lack of peripheral mononuclear cells present in the diseased brain further suggests that neurodegeneration is not driven by the peripheral immune response, but rather by the presence of chronically activated, pro-inflammatory microglia that were generated in response to neuronal dysfunction, or from changes within their genetic make-up [44, 45].

The potential neurotoxic consequences of chronically activated, pro-inflammatory microglia have remained a topic of intensive research in recent years, and whether skewed microglial responses can directly promote the development of neurodegenerative disease is still inconclusive. However, considerable evidence suggests that chronically activated microglia continually secrete neurotoxic molecules to sustain neuroinflammation. This is supported by histological and biochemical studies demonstrating that numerous pro-inflammatory molecules are increased in both the serum and cerebrospinal fluid in the context of neurodegenerative diseases and head injuries [47–52]. Pro-inflammatory cytokines can have neurotoxic effects by triggering intracellular apoptotic pathways in neurons through activation of caspase-3 and caspase-9, promoting oxidative stress via the release of cytochrome C, and by inducing excitotoxicity through augmentation of Ca^2+^ ion sensitivity and influx [53–55].

Despite the seemingly prominent role of microglia in the onset of chronic neuroinflammation, the mechanisms underlying the transition towards a persistent, deleterious response are still unclear. To date, multiple factors have been speculated to induce abnormal microglial activation, including aging, peripheral infection, systemic inflammation, as well as genetic predispositions and mutations [56, 57]. Fundamental research to identify and map the various intracellular signal transduction pathways that are involved in regulating microglial responses and polarization is evidently required, to better understand the role of signaling cascades, such as the PI3K-AKT pathway. Recent studies have implicated this pathway in both microglial responses as well as neurodegeneration in the context of Alzheimer’s disease and Parkinson’s disease [21, 58, 59].

### PI3K-AKT signaling pathway: the basics

**Fig. 1:**
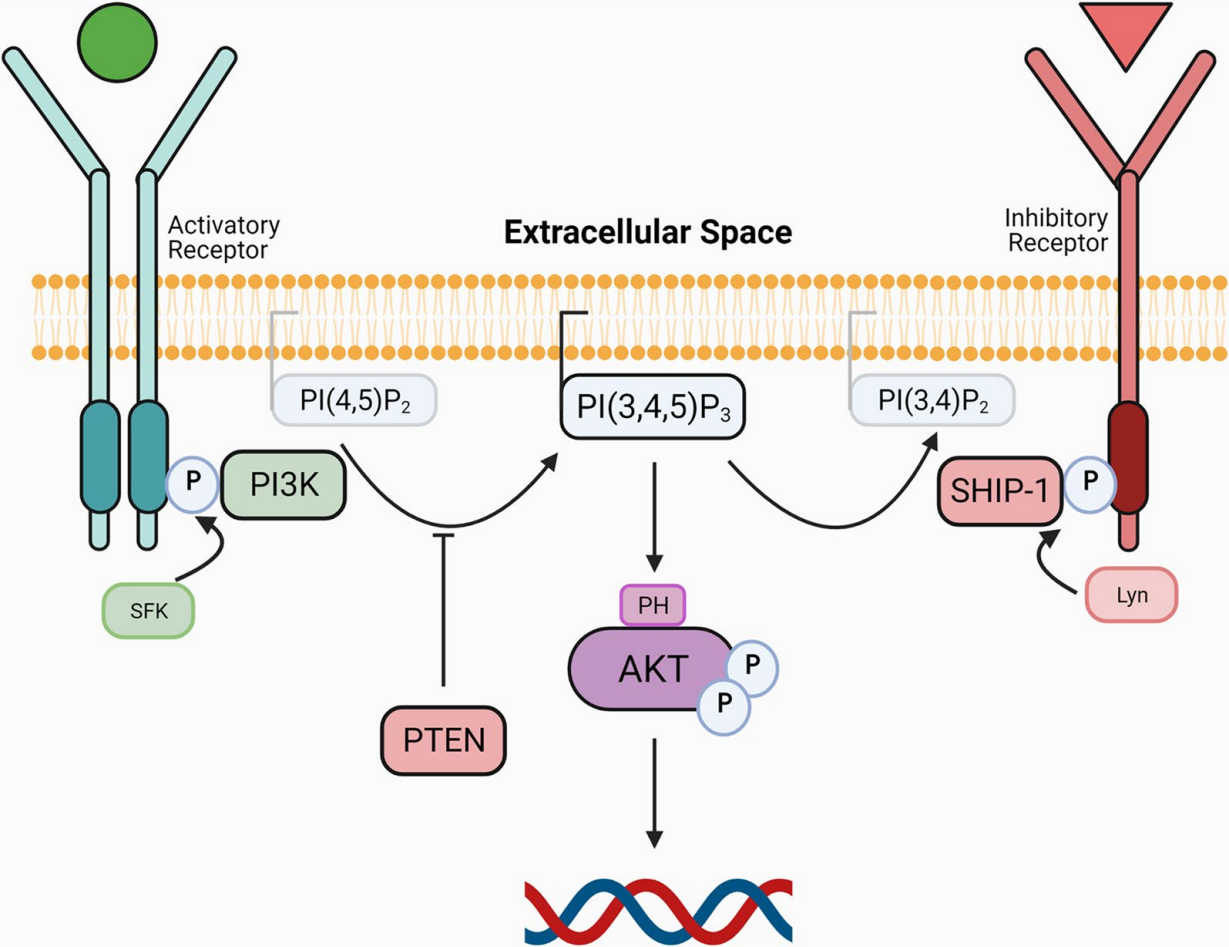
PI3K-AKT signaling pathway in microglia. PI3K is comprised of a p85 regulatory subunit that directs its location and a p110 catalytic subunit that phosphorylates phospholipids to mediate downstream signaling. In response to activation signals, PI3K is recruited to phosphotyrosine-containing motifs in cell surface receptors via the SH2 domain in p85, whereupon it acts on the membrane phospholipid, PI(4,5)P_2_, generating the second messenger PI(3,4,5)P_3_. PI(3,4,5)P_3_ recruits protein kinase B (AKT) to the membrane resulting in its activation, where it can phosphorylate numerous downstream substrates, leading to changes in gene transcription and biological responses. Upon receiving an inhibitory signal, the lipid phosphatase SHIP-1 is recruited to the membrane, where it converts PI(3,4,5)P_3_ into phosphatidylinositol 3,4 bisphosphate (PI(3,4)P_2_) to downmodulate signaling. PTEN is a direct antagonist of PI3K, converting PI(3,4,5)P_3_ to PI(4,5)P_2_. *SFK* Src family tyrosine kinase; *Lyn* Lck/yes-related novel tyrosine kinase; *PH* Pleckstrin homology domain

Phosphatidylinositol 3-kinases (PI3K) are a family of intracellular lipid kinases that transduce signals from cell surface receptors, such as receptor tyrosine kinases, receptors that lack intrinsic tyrosine kinase activity, and heterodimeric G protein-coupled receptors [60]. Upon ligand binding, the receptors become tyrosine phosphorylated on specific motifs in their intracellular domain, either via their own intrinsic catalytic activity or through the action of non-receptor tyrosine kinases. This induces recruitment of PI3K to these sites, where it is then localized to its substrate, the abundant membrane phospholipid phosphatidylinositol 4,5 bisphosphate (PI(4,5)P_2_). PI3K phosphorylates PI(4,5)P_2_ to generate the key second messenger phosphatidylinositol 3,4,5 trisphosphate (PI(3,4,5)P_3_), which recruits the intracellular signaling molecule AKT (also commonly known as protein kinase B (PKB)), a serine/threonine-specific protein kinase (Fig. 1). This leads to AKT activation, whereupon it phosphorylates target proteins, leading to changes in cellular responses. This pathway is tightly regulated via phosphorylation and dephosphorylation of various enzymes, adaptor proteins, and phospholipids (for review see [61, 62]). Two lipid phosphatases that negatively regulate this pathway are phosphatase and tensin homolog (PTEN) and SH2-domain-containing inositol 5’ phosphatase-1 (SHIP-1), which convert PI(3,4,5)P_3_ to PI(4,5)P_2_ or PI(3,4)P_2_, respectively (Fig. 1).

Abundant evidence demonstrates a central role for the PI3K-AKT signaling pathway in health and disease. Activated AKT is able to phosphorylate and activate a range of substrates in the cytoplasm and nucleus that mediate numerous cellular functions critical for survival, proliferation, differentiation, migration, and glucose uptake and metabolism [63–66]. In addition, activated AKT is vital for initiating immune responses, as evidenced by the functional defects observed in various leukocyte subsets when PI3K and AKT are ablated in experimental rodent models [67, 68]. However, unchecked activation of this pathway has detrimental effects; hyperactivity of this pathway is a major contributor to oncogenesis, as well as the exacerbated immunological responses and persistent inflammation that underlie various systemic diseases (for review see [60, 69]).

Similarly, in the brain, dysregulation of the PI3K-AKT signaling pathway is involved in numerous pathologies, such as neurodegenerative diseases and brain tumor metastasis [58, 70]. However, in contrast to other biological systems, our understanding of its function in the neuroimmune system is still limited. Considering the known role of this pathway in mediating inflammatory responses outside of the brain, dysfunctional signaling in PI3K-AKT is also likely to contribute to the onset and perpetuation of neuroinflammation [20, 62]. PI3K-AKT signaling in the brain appears to be intimately linked with microglial activity and activation, with many of the key regulatory receptors that activate PI3K-AKT pathway being expressed by microglia [62]. Activation of these receptors stimulates PI3K-AKT signaling and induces microglial activation and cytokine production, therefore, implying that abnormalities in PI3K-AKT signaling may drive a pro-inflammatory microglial phenotype that contributes to a wide-range of diseases and disorders [62].

## Aberrant PI3K-AKT signaling in microglia as a driver of chronic neuroinflammation

### PI3K-AKT activation shapes microglial inflammatory responses

The role of PI3K-AKT signaling in modulating microglial activity and inflammatory responses was first observed in in vitro studies almost two decades ago. Stimulating mouse BV-2 microglial cells with the anti-inflammatory cytokine transforming growth factor-beta resulted in a reduction of PI3K-AKT-driven nitric oxide (NO) production and improvement in cell survival [71]. Conversely, neurotoxic plaques were found to stimulate PI3K-AKT signaling in primary neonatal mouse microglia and Ra2 microglial cell lines, resulting in changes in their production of pro-inflammatory chemokines [72]. Since then, PI3K-AKT signaling has been recognized as the central regulator of microglial activity in response to extrinsic stimuli including LPS and inflammatory cytokines [73, 74]. For example, in vivo systemic challenge with LPS in rodents leads to an increase in phosphorylation of AKT via PI3K signaling in the CNS, which induces activation of microglial populations in the cortex, hippocampus, and thalamus, and is associated with exacerbated release of the pro-inflammatory factors TNF-α, IL-1β, iNOS, and IL-6 [75, 76]. While these findings suggest that LPS stimulation of microglia promotes a predominantly pro-inflammatory response, it is important to note that LPS can enter the CNS across a compromised blood brain barrier in states of injury or disease, and activate other glial cells as well as the peripheral immune response, which likely also contributes to the neuroinflammatory phenotype exhibited by these mice [77].

On the contrary, PI3K-AKT signaling may also promote an anti-inflammatory response in microglia, whereby activation of AKT may be neuroprotective. A recent study by Bhat and colleagues found that use of an anti-inflammatory modulator that promoted AKT activity in an experimental TBI model resulted in decreased pro-inflammatory microglial activation and favorable outcomes in rodents [78]. In addition, activation of AKT in primary human fetal microglia after LPS stimulation has been reported to enhance expression of the IL-1β receptor, IL-10 and interferon-β while also dampening the pro-inflammatory response [74]. Of note, some of the opposing findings in the literature, particularly from in vitro studies, may be attributed to distinct signaling pathways for activation of inflammatory responses in fetal versus adult cell types, which requires further clarification [79, 80]. Several studies have identified age-related changes in glial responses and neuroinflammatory profiles in rodents that are likely driven by changes in PI3K-AKT signaling in the aging brain. For example, ex vivo analysis of microglial populations collected from young and aged rats identified an age-dependent change in their function, such as increased pro-inflammatory cytokine production and reduced phagocytic abilities [81]. Such findings are supported by in vivo work in which NF-κB activation and TNF-α levels in the rodent hippocampus were increased in an age-dependent manner, alongside a reduction in AKT activation and an increase in glycogen synthase kinase-3β activity [82]. While age-related changes to microglial activity remain poorly understood, it is plausible that PI3K-AKT signaling variations in the aging brain skews the microglial response towards a pathogenic state, which may underlie the development of several neurodegenerative diseases.

Collectively, these studies suggest that activation of the PI3K-AKT signaling cascade in microglia within an in-vivo setting can induce both arms of the inflammatory response. However, it appears that the M1/M2-like polarization of microglia is influenced by multiple factors, and more detailed characterization of activated microglia is required.

### Key cell surface receptors that direct microglial responses via PI3K signaling

**Fig. 2:**
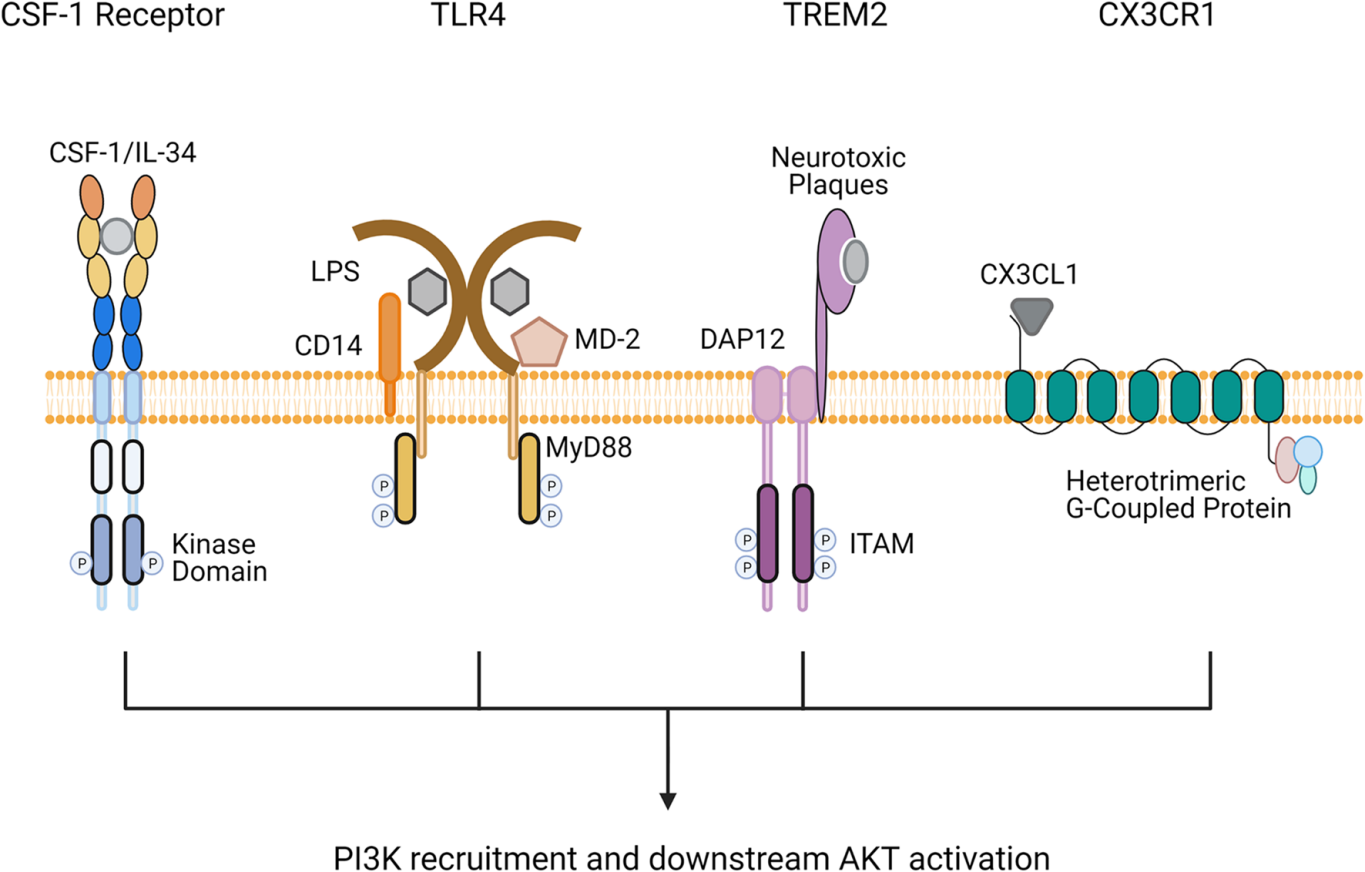
Key cell surface receptors in microglia that utilize PI3K-AKT signaling. CSF-1 or IL-34 binds to CSF-1R on microglia, triggering its dimerization, autophosphorylation and recruitment of PI3K. Endotoxin (LPS) binds to the TLR4 complex comprised of CD14 and MD-2, causing it to dimerize and induce recruitment of the adaptor MyD88, leading to its phosphorylation and the promotion of PI3K signaling. Neurotoxic plaques or other agonists bind to TREM2 and the co-associated membrane-bound adaptor protein DAP12 becomes phosphorylated on tyrosine-containing activation motifs (ITAMs), resulting in PI3K recruitment and activation. CX3CL1 binds to CX3CR1 and triggers the activation of heterotrimeric G proteins that transduce an intracellular signal via PI3K

A number of essential microglial cell surface receptors utilize the PI3K-AKT signaling network, with signaling from these receptors directing microglial responses both during homeostasis and neuroinflammatory disease (Table 1) (Fig. 2) [73, 83–85]. The following section will describe four of these key receptors in more detail and indicate, where their aberrant expression or activity has been implicated in various neurological disorders.

**Table 1:** Key cell surface receptors on microglia that utilize PI3K-AKT signaling

Receptor	Ligands	Disease implication	Key references
CSF-1 receptor	CSF-1, IL-34	Reduced signaling: blunted brain development, leukoencephalopathy, olfactory deficits Increased signaling: progressive MS, AD	[86–90]
CX3CR1	CX3CL1	Reduced signaling: PD, ALS	[91]
Fibroblast growth factor receptor 2	Fibroblast growth factor	Reduced signaling: MS	[92]
IL-4 receptor	IL-4	Reduced signaling: AD, MS	[93, 94]
Insulin receptor	Insulin	Reduced signaling: AD	[95, 96]
TLR4	LPS, LTA	Increased signaling: AD, PD, ALS	[97–99]
TREM2	LPS, bacterial products, DNA, phospholipids, lipoproteins, sulfatides, Aβ plaques	Reduced signaling: AD, Nasu-Kakola disease	[100–103]

*Aβ plaques* amyloid beta plaques, *AD* Alzheimer’s disease, *ALS* amyotrophic lateral sclerosis, *CSF-1* colony stimulating factor 1, *CX3CL1* C–X3–C motif ligand 1, *DNA* deoxyribonucleic acid, *IL* interleukin-, *LPS* lipopolysaccharide, *LTA* lipoteichoic acid, *MS* multiple sclerosis, *PD* Parkinson’s disease, *TLR* Toll-like receptor, *TREM*2 triggering receptors expressed on myeloid cells 2

#### Colony-stimulating factor 1 receptor: a key regulator of microglial development

Colony-stimulating factor 1 receptor (CSF-1R), also known as macrophage colony-stimulating factor receptor (M-CSFR) or CD115, is a type 1 membrane protein that is critical for the development, differentiation, survival, and function of monocytes and tissue macrophages [104]. In the brain, CSF-1R is primarily expressed by microglia, and microglial development and maintenance at pre- and post-natal stages is largely dependent on CSF-1R signaling [105, 106]. The CSF-1R has two ligands, CSF-1 and the more recently identified IL-34, which have overlapping but also distinct functions in macrophage homeostasis. Induction of CSF-1R signaling with either CSF-1 or IL-34 has a proliferative effect, and while both similarly induce activation of Akt in human monocytes and their differentiation, they promote different macrophage polarization and cytokine/chemokine responses [107]. Nonetheless, CSF-1 has been shown to directly promote the polarization of microglia towards a regenerative phenotype in mouse brain, and previous studies in macrophages suggest that this mechanism occurs via activation of the PI3K-AKT signaling cascade [108–112]. Recently, it was reported that engagement of the CSF-1R receptor on microglia results in its oligomerization and phosphorylation of a tyrosine residue necessary for PI3K recruitment; suggesting that CSF-1R signaling in microglia is indeed dependent on the PI3K-AKT pathway in this context (Fig. 2) [113, 114].

Abnormal CSF-1R expression is thought to interfere with microglia functioning and contribute to neurodevelopmental disorders and neurodegenerative diseases [115]. Indeed, hereditary loss or mutations within the *Csf1r* gene ablated microglial development and proliferation, resulting in congenital absence of microglia in the developed brain, which resulted in neurological and other developmental abnormalities [86]. Blunted brain development was also reported in *Csf1r−/−* mice and in mice with a loss-of-function mutation in the gene encoding CSF-1 (*Csf1*^*op/op*^), with *Csf1*^*op/op*^ mice displaying pronounced postnatal olfactory deficits [87, 88]. Surprisingly, the density of microglia in the brains of mice *Csf1*^*op/op*^ mice appeared to be unchanged compared to mice harboring a homozygous null mutation in *Csf1r* [116, 117]. This finding suggested that CSF-1 was not the sole ligand for CSF-1R, and indeed, it is now known that IL-34 is another binding partner for CSF-1R [118].

Increased CSF-1R expression and CSF-1 levels within the brain have recently been identified as mediators of demyelination in progressive multiple sclerosis by perpetuating microglial survival and proliferation to exacerbate neuroinflammation [89]. Inhibiting CSF-1R signaling in an experimental rodent model of progressive multiple sclerosis attenuated microglial survivability and pro-inflammatory responses, further suggesting that CSF-1R signaling can influence microglial phenotype [89]. Furthermore, deletion of CSF-1R in the APP/PS1 mouse model of Alzheimer’s disease ameliorated cognitive decline and plaque volume in the cortex and hippocampus [90]. Although these studies were the first to identify a neuropathogenic effect associated with elevated CSF-1R signaling, future investigations may reveal pathological consequences related to increased CSF-1R signaling in other neurodegenerative diseases. Nonetheless, taken together, these studies indicate that aberrant PI3K-AKT signaling due to abnormal CSF-1R expression is detrimental to brain development, and may promote the onset of neurodegenerative diseases.

#### Toll-like receptor 4: an important mediator of microglial activation

Toll-like receptors (TLR) are a distinct class of receptors expressed on an array of innate immune cells, where they recognize conserved molecular patterns in microbes. Several subclasses of TLRs have been identified on microglia, including TLR2, TLR3, TLR4, TLR5, TLR7, and TLR9 [119]. TLRs can interact with various immunostimulants, such as endotoxins, which induce microglial activation and subsequent inflammatory responses [119, 120]. TLR4, which recognizes bacterial LPS by forming a complex with co-receptor CD14 and myeloid differentiation factor 2 (MD-2), is perhaps the best-studied of the TLRs, with numerous analyses implicating TLR4-dependent microglial activation in various scenarios of neuroinflammation and neurodegenerative diseases [121, 122].

Most TLRs, including TLR4, signal via the cytosolic adaptor protein myeloid differentiation primary response 88 (MyD88). The binding of LPS to the TLR4 complex leads to the tyrosine phosphorylation of MyD88, which induces recruitment of PI3K via its p85 domain leading to its subsequent activation (Fig. 2) [123, 124]. The exact downstream signaling mechanism following MyD88 recruitment in microglia is still unclear; but in human macrophages and myeloid cells, the coupling between a phosphorylated YXXM motif in MyD88 and PI3K induces PIP_2_ conversion, in turn causing AKT phosphorylation at serine 473 and leading to the recruitment of NF-κB [124].

Several studies have provided evidence that activation of microglia through TLR4 occurs via the PI3K-AKT pathway [85, 125]. For example, TLR4 activation following spinal cord injury in experimental rodents induced microglial pyroptosis through activation of the PI3K-AKT pathway [85]. Ex vivo studies also reflected a similar relationship: one study in chicken embryonic microglia reported that PI3K-AKT signaling was required for LPS-TLR4-dependent activation of microglia [73]. Furthermore, this study found that inhibiting AKT phosphorylation can mitigate the LPS-induced responses [73]. Similarly, a recent study found that inhibition of TLR4 activity in BV-2 microglia prior to LPS stimulation reduced phosphorylated AKT levels compared to untreated cells, resulting in reduced viability and cytokine production [126]. Recently, a study by Xu et al. noted that interactions between TLR4 and pathogenic protein aggregates on BV-2 microglia stimulated the PI3K-AKT signaling cascade which resulted in a pro-inflammatory response [85]. Together, these studies suggest that LPS-TLR4 activation in microglia induces PI3K-AKT signaling.

Abnormal TLR4 expression has been implicated in impaired microglial functioning and neurodegenerative diseases, presumably via its link to PI3K activity. Endogenous TLR4 expression is thought to be neuroprotective in some contexts; for example, by mediating microglial autophagy of neurotoxic plaques in primary mouse microglia and experimental rat models of Parkinson’s disease [127, 128]. Conversely, over-expression or chronic activation of TLR4 on microglia may contribute to pathogenesis and neurodegeneration. Indeed, increased TLR4 expression has been identified in the brains of patients with Alzheimer’s disease, while persistent TLR4 stimulation in rodent models of Alzheimer’s disease exacerbates disease burden [97]. Moreover, increased TLR4 expression has been detected in the substantia nigra of patients with Parkinson’s disease, and in a mouse model of amyotrophic lateral sclerosis [98, 99]. It is speculated that increased TLR4 expression on microglia inhibits their anti-inflammatory phenotype while simultaneously prolonging their pro-inflammatory response [129]. This hypothesis is further supported by the pronounced reduction in neuroinflammation in a rodent model of Parkinson’s disease following TLR4 ablation [130, 131]. Taken together, increased TLR4 expression or continual TLR4 stimulation, and subsequent PI3K-AKT activation, perpetuates neuroinflammation by stimulating microglial activation.

#### TREM2: a duplicitous receptor involved in both microglial activation and inhibition

Triggering Receptor Expressed on Myeloid Cells 2 (TREM2) is a transmembrane receptor of the immunoglobulin superfamily that was initially identified in monocyte-derived dendritic cells and mouse macrophages [132]. TREM2 can bind to an array of anionic ligands including phospholipids, lipoproteins, sulfatides, bacterial LPS and DNA, as well as various forms of amyloid-β, and it interacts with the transmembrane region of the adaptor protein DNAX-activation protein 12 (DAP12) to facilitate signal transduction [133] (Fig. 2). In bone marrow-derived macrophages and monocytes, TREM2 ligation results in phosphorylation of DAP12 on tyrosine residues in activation motifs via Src family kinases leading to the recruitment of Syk tyrosine kinase and the downstream activation of PI3K and AKT [134–137]. In microglia, targeted overexpression of TREM2 in the mouse BV-2 cell line correlated with an increase in AKT activity and microglial phagocytosis [137]. In agreement with these studies, depletion of TREM2 in mice reduced AKT activity in microglia, resulting in arrested cell cycling and reduced cell survival [138]. Moreover, in an Alzheimer’s disease model, deficiency of TREM2 in microglia was associated with reduced phosphorylation of AKT and poor activation of the downstream transcription factor mTORC1, leading to altered metabolic pathways, cell death, and augmented neuronal dystrophy [135]. In contrast, a recent study has correlated TREM2 overexpression in BV-2 microglia with reduced AKT and NF-κB phosphorylation after LPS activation, suggesting that overexpression of TREM2 inhibits PI3K activation in reactive microglia [136]. Collectively, the above findings suggest that TREM2 regulates the PI3K-AKT signaling cascade in microglia; however, conflicting results show that TREM2 expression can both promote and suppress AKT activity, which may reflect the context in which it is activated.

Emerging evidence has suggested that abnormal TREM2 expression and signaling within the brain may promote progressive dementia, such as Alzheimer’s disease and Nasu–Hokola disease, which is a genetic disorder characterized by early-onset dementia [100]. Genome sequencing of patients with Alzheimer’s disease and Nasu–Hokola disease has correlated *Trem2* mutations with microglial disorders and disease progression [101–103]. Interestingly, two of the common TREM2 variants observed in Alzheimer’s disease can bind to amyloid-β similarly to TREM2 but fail to trigger amyloid-β internalization and exhibit reduced signaling responses [139]. Similarly, genetic analyses of Nasu–Hakola patients has revealed that they express mutant variants of TREM2 that are non-functional, but the exact molecular mechanisms underlying this disease is still inconclusive [140]. Recently, it was reported that TREM2 acts as a receptor for neurotoxic oligomers, and interactions between the receptor and ligand mediates microglial responses, which in turn accelerate the elimination of these pathogenic plaques [141, 142]. Genetic ablation of TREM2 expression on microglia in experimental rodents impaired their phagocytic ability and inhibited their ability to release both pro- and anti-inflammatory cytokines, resulting in increased susceptibility to neuron dystrophy and plaque seeding [141, 142]. Although prolonged release of pro-inflammatory cytokines is often considered neuropathological, transient secretion of both classes of inflammatory cytokines have previously demonstrated neuroprotective effects against neurotoxic plaques in Alzheimer’s disease [143]. Therefore, it is speculated that reduced expression of TREM2 significantly impairs plaque clearance and pro-inflammatory responses, likely resulting from limited AKT activation, with consequences for disease progression.

#### CX3CR1: a mediator of crosstalk between microglia and neurons

CX3CR1, also known as fractalkine receptor or G-protein-coupled receptor 13, is a membrane-bound chemokine receptor expressed on most early myeloid lineage cells, which mediates their adhesion and migration in response to CX3CL1, which is mostly expressed by neurons [144]. In the brain, *Cx3cr1* is one of the most highly expressed genes in human and mouse microglia, and activation of CX3CR1 on microglia induces the PI3K-AKT signaling cascade in a dose-dependent manner [145, 146]. Since CX3CR1 triggers AKT activation, its expression is implicated in several critical responses of microglia to injury and inflammatory stimuli, including cell activation, cytokine production, migration, and phagocytosis [146, 147].

Increasing evidence indicates that disruption to the CX3CR1 signaling pathway can produce disease-specific microglial responses that promote neuropathology in various brain disorders. Perhaps unexpectedly, an initial study reported that ablation of CX3CR1 signaling in microglia led to an exacerbated rather than attenuated microglial response, significant neurotoxicity to a peripheral LPS challenge, as well as worsened neurodegeneration in experimental models of Parkinson’s disease and amyotrophic lateral sclerosis [91]. These effects were largely attributed to the extended microglial activation and prolonged release of the pro-inflammatory cytokine IL-1β, which increases glutamate uptake on synapses and triggers excitotoxicity [39, 91]. Moreover, CX3CR1 deficiency has also been linked to elevated pro-inflammatory microglial markers and pronounced cognitive deficits after mild head trauma [148]. Based on these studies, one may speculate that reduced CX3CR1 and AKT signaling in microglia skews their responses towards a pro-inflammatory phenotype. In line with this, increased CX3CR1 signaling was found to be neuroprotective by reducing microglial activation in experimental models of Parkinson’s disease models and proteinopathies [149, 150]. These findings highlight that abnormal PI3K-AKT signaling can produce nonconventional microglial responses that may be either pathologic or beneficial, indicating that the regulatory role of PI3K-AKT within microglial responses is highly dynamic and complex.

Overall, numerous studies have shown that PI3K-AKT is a key signaling pathway utilized by microglia for regulating their response to various extracellular stimuli. This pathway not only underpins microglial development and activation, but is central for maintaining brain homeostasis. Abnormal PI3K-AKT signaling as a result of dysregulation in specific cell surface receptor expression or functionality disrupts microglial activities thereby promoting neurological disease.

#### Aberrant PI3K-AKT signaling in neurological disorders and diseases

**Fig. 3:**
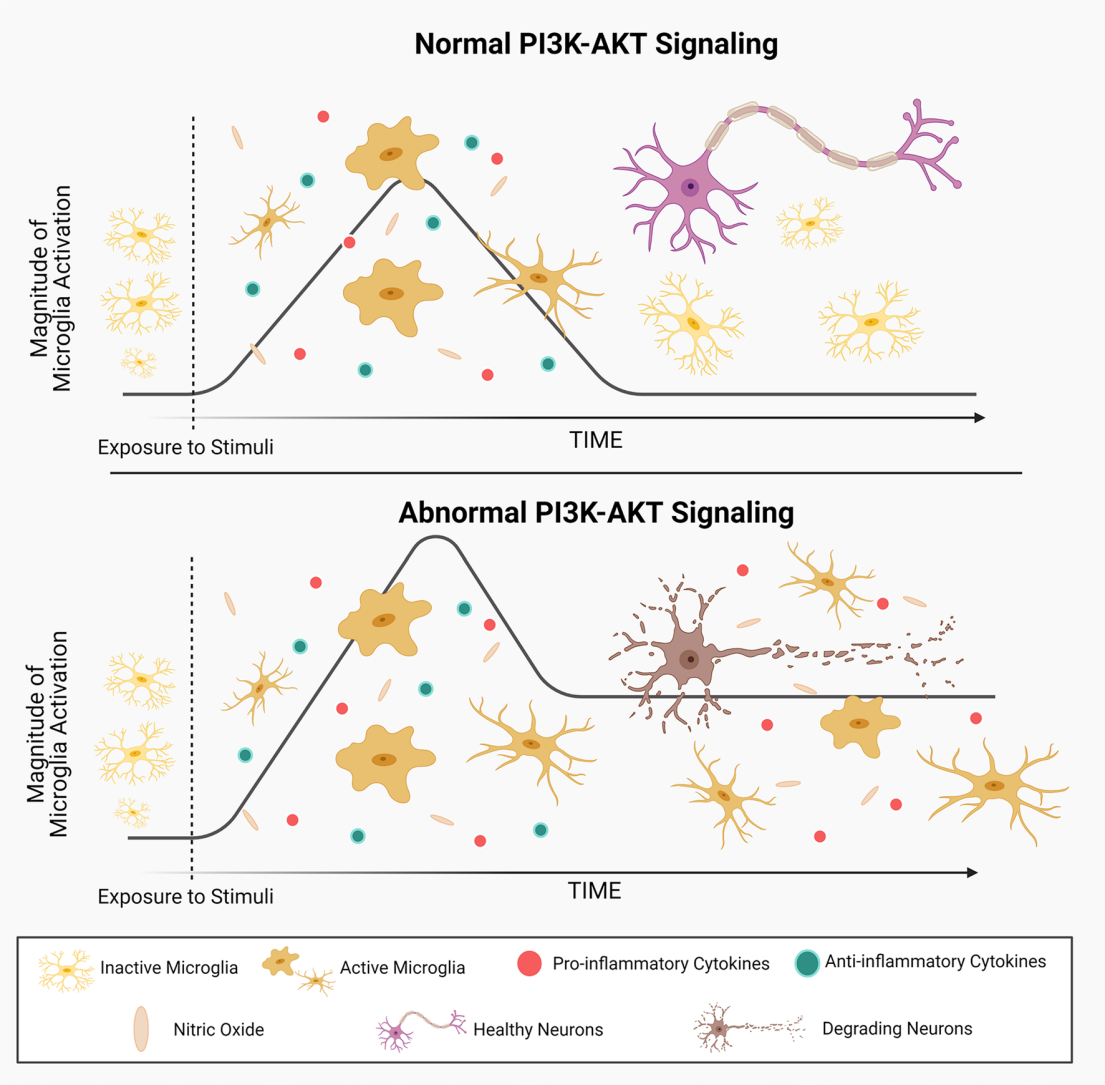
Dysregulation of PI3K-AKT signaling causes abnormal microglial responses and promotes neuronal damage and chronic neuroinflammation. Resting microglia undergo activation upon encountering stimuli in their environment, triggering the release of nitric oxide and the coordinated expression of pro- and anti-inflammatory cytokines to clear the stimulus and promote recovery. Under normal conditions, PI3K-AKT signaling is terminated, the microglial response subsides and neurons are preserved. However, in certain settings, PI3K-AKT signaling is sustained, microglial responses persist perpetuating the release of pro-inflammatory cytokines into the brain microenvironment, which damages healthy neurons and contributes to progressive neurodegeneration

Based on the demonstrated role of PI3K-AKT signaling in the regulation of microglial responses and inflammatory phenotype, it is safe to hypothesize that disruption to this pathway promotes chronic microglia-driven neuroinflammation and neurodegeneration (Fig. 3). As outlined in Table 2, emerging evidence of dysregulated PI3K-AKT signaling in various disease states provides support for this theory.

**Table 2:** Dysregulated PI3K-AKT signaling in neurodegenerative diseases

Disease	Signaling defects	Study type	Outcomes	Key references
Alzheimer’s disease	Polymorphic mutations within *INPP5D*, presumably altering negative regulation of PI3K-AKT signaling	Clinical	Risk factor for late-onset AD Associated with disease-related neuropathology	[151–154]
		Preclinical—mouse	Reduced plaque uptake by microglia	[155]
Huntington’s disease	Elevated AKT proteins in lymphoblasts and *Akt1* in monocytes	Clinical	Associated with HD development Increased IL-1β and TNF-α levels Microgliosis	[156, 157] [158]
		Preclinical—mouse	Increased IL-1β and TNF-α levels Microgliosis	[159]
Parkinson’s disease	Abnormal *PINK1* activity, altering PI3K-AKT signaling	Clinical	Recessively inherited form of PD	[160, 161]
		Preclinical—mouse	Altered AKT activity in microglia and induces a pro-inflammatory response	[162, 163]

*AD* Alzheimer’s disease, *AKT* protein kinase B, *HD* Huntington’s disease, *IL-1β* interleukin 1 beta, *INPP5D* inositol polyphosphate-5-phosphatase D, *PD* Parkinson’s disease, *PI3K* phosphoinositide 3-kinase, *PINK1* PTEN-induced serine/threonine–protein kinase 1, *TNF-α* tumor necrosis factor-alpha

The relationship between PI3K-AKT dysregulation and neurodegenerative disease has been most prominently explored in the context of Alzheimer’s disease. Several genome-wide association studies have indicated that single nucleotide polymorphic (SNP) variants of the SHIP-1 encoding gene, *INPP5D*, are an Alzheimer’s disease risk factor and they are associated with Alzheimer’s disease-related neuropathology in humans [151–153, 164]. Transcriptomic analyses demonstrated that these variants are conserved between humans and mice, while ex vivo analysis found that Alzheimer’s disease-related SNPs increase SHIP-1 expression in myeloid cells [151, 165]. Interestingly, several studies have correlated increased plaque deposition to increased *Inpp5d* expression in humans and animal models, therefore, suggesting that disease-related SNPs impair the functionality of SHIP-1 but not its expression, which may in turn enhance PI3K-AKT signaling to exacerbate microglial responses during the early stages of Alzheimer’s disease development [151, 154, 155]. Furthermore, SHIP-1-deficient mice have augmented PI3K/AKT signaling and are prone to developing progressive lung inflammation as a result of exacerbated immune responses and enhanced macrophage activation in lung airspaces [166]. Therefore, abnormalities in SHIP-1 activity within the brain may elicit pathogenic microglia responses that drive or contribute to the development of Alzheimer’s disease [167]. Collectively, these findings indicate that dysregulation in AKT activity in microglia, brought about by defective negative regulation through changes in *INPP5D*, contributes to the development of Alzheimer’s disease.

Similarly, dysregulation of PI3K-AKT signaling has also been linked to the development of Parkinson’s disease. Abnormal expression of the PTEN-induced serine/threonine–protein kinase 1 (PINK-1) or mutations in the *PINK1* gene are linked to the recessively inherited form of Parkinson’s disease [160, 161]. As a downstream substrate of PTEN, PINK1 is a vital regulator of inflammatory responses during stress, and directly activates PI3K in mitochondria [168]. Increased microglia and astrocyte reactivity and cell number were observed in myelinated brain regions of *Pink1*^*−/−*^ mice, likely in response to debris from neurons undergoing mitophagy [169]. More recently, PINK1 was found to be ubiquitously expressed in primary rat microglia; and in experimental mouse models of Parkinson’s disease, reduced PINK1 diminishes AKT activity in microglia [162, 170]. Interestingly, mixed primary *Pink1*^*−/−*^ mouse astrocyte and microglia cultures exhibited elevated NO production and TNF-α and IL-1β transcripts in response to LPS, IFN-γ and alpha-synuclein proteins, relative to primary glial cells from wild type mice [163]. The underlying relationship between reduced PINK1 in microglia and the pro-inflammatory response is unclear, but PINK1 has been found to accumulate within mitochondria of RAW 267.4 macrophages in response to LPS and facilitate clearance of dysfunctional mitochondria [171]. However, in the same study, knocking down PINK1 resulted in accumulation of dysfunctional mitochondria in macrophages following LPS stimulation and elevated their pro-inflammatory cytokine production [171]. Therefore, abnormal PINK1 activity in microglia appears to result in increased dysfunctional mitochondrial accumulation and apoptosis, leading to the release of oxidized mitochondrial DNA, which triggers the activation of inflammasomes and cytokine release [171, 172].

The PI3K-AKT signaling pathway has also been implicated in the etiology of Huntington’s disease. Activation of this pathway in neurons via insulin growth factor 1 is protective against huntingtin-induced toxicity in experimental Huntington’s disease models; however, contrasting observations were made in the peripheral immune cell compartment of Huntington’s disease patients [173]. Post-mortem analysis of limited blood samples from Huntington’s disease patients has found elevated AKT protein levels in their lymphoblasts, and elevated *Akt1* has been documented in monocytes generated from experimental disease models prior to disease development and during neurodegeneration, therefore, correlating changes in AKT activity to disease onset [156, 157]. Interestingly, analysis of lymphoblasts collected from Huntington’s disease patients revealed that AKT is cleaved by caspase 3, the frequently activated death protease, during late stages of disease development [157]. Currently, the exact role of caspase 3 in microglia is unclear, although it has been speculated that deactivation of AKT via caspase 3 is a protective mechanism against irregular activation of microglia to prevent neuroinflammation. However, contrasting evidence indicates that deactivation of caspase 3 and caspase 7 in BV2 microglia reduced their activation and cytokine production in response to proinflammogens [159, 174, 175]. It is important to note that activation of caspase 7 leads to microglial activation, which could explain the contrasting findings [176]. Nonetheless, increased activity of the AKT substrate NF-κB has since been recognized as a hallmark of Huntington’s disease. Furthermore, both elevated AKT and NF-κB activities have been correlated with increased levels of pro-inflammatory cytokines, such as IL-1β and TNF-α, as well as marked microgliosis both in patients and experimental rodents, therefore, suggesting that unchecked AKT activity from abnormal caspase activity could induce pathogenic microglial responses [158]. Thus, a link has also been established between dysregulated PI3K-AKT signaling in microglia and neuropathology during the development of Huntington disease.

However, the current paradigm of PI3K-AKT signaling in neurodegenerative diseases is primarily based on in vitro and ex vivo work using both microglia and macrophages, and experimental rodent models. Therefore, further clinical studies of microglial responses in neurological disease settings are required to provide greater insight into how alterations in PI3K-AKT signaling induces a pathogenic state in microglia.

### Modulating the PI3K-AKT pathway as a therapeutic approach

With evidence mounting in support of a central role for PI3K-AKT signaling in neurodegenerative disease, it is feasible that modulation of this pathway may be a means to control disease pathogenesis. The readers are directed to an excellent review that summarizes specific compounds that have been utilized to modulate PI3K activity in the brain [62]. To date, the primary target for modulation of the PI3K-AKT signaling pathway in microglia involves reducing or inhibiting PI3K translocation to the cellular membrane. Inhibition of PI3K localization with lithium following LPS stimulation of mouse BV-2 microglial cells, was shown to revert their classical pro-inflammatory phenotype and reduce IL-6 and TNF-α production [177]. In addition, extrinsic inhibition of PI3K signaling with the immunosuppressive drug, 6-mercaptopurine, produced a neuroprotective effect by impeding the production of TNF-α in LPS-activated BV-2 microglia [178]. In another study, the application of morin, a flavonoid extracted from certain fruits and herbs, was found to induce anti-inflammatory responses in both BV-2 microglia and in mice after LPS stimulation, by downregulating PI3K-AKT signaling [179]. Finally, pre-treating BV-2 microglia with the potent PI3K inhibitor LY294002 prior to LPS stimulation significantly reduced AKT activation, which subsequently reduced viability, and switched their phenotype from pro-inflammatory to anti-inflammatory [136]. Together, these studies emphasize the potential for therapeutics that modulate the PI3K-AKT pathway in microglia to dampen pathological responses and induce neuroprotection in experimental rodents*.*

Targeting PI3K in in vivo disease and injury models has likewise revealed promising neuroprotective effects. Inhibition of PI3K activity in 3–6-month-old mice in an Alzheimer’s disease model dampened microglial secretion of TNF-α, and consequently increased survival and prevented cognitive decline in comparison to non-treated mice [180]. In addition, utilization of the anti-PI3K compound ZsTK474 in animals following experimental stroke blunted gene expression of *Tnf-α*, *Il-1β* and *Il-6* in the injured brain and induced a doubling of anti-inflammatory factors during the first 48 h after injury, while neuronal damage was halved compared to non-treated stroke animals [181]. In other studies, suppression of NF-κB signaling downstream of AKT reduced the pro-inflammatory polarization of microglia in rodent models of neurodegenerative disease while simultaneously mitigating neurodegeneration and behavioral anomalies [182, 183]. In line with this, endurance exercise in a Parkinson’s disease rodent model rescued the neuroinflammatory-dominant phenotype in microglia by suppressing intracellular NF-κB signaling [184]. Together, these studies are promising for future therapeutic targeting of PI3K-AKT in the clinical setting. However, more research investigating the long-term effects of PI3K inhibition on chronic disease and injury-induced inflammation is still needed, particularly since these inhibitors may adversely impact the normal functioning of the immune system.

While there is strong support to indicate that PI3K inhibition may be a therapeutic approach in certain neurological disease settings, there is also evidence to suggest that induction of PI3K-AKT signaling, through the use of anti-inflammatory factors, may be required in some disease contexts. The upregulation of PI3K-AKT signaling following exogenous application of the anti-inflammatory mediator fibroblast growth factor 10 in mice following experimental spinal cord injury, reduced IL-6 and TNF-α production and promoted an anti-inflammatory skewed microglial response [185]. In another study, the application of electrical therapy in rats after ischemic stroke, which exerts a typical anti-inflammatory effect, upregulated AKT activity in the ischemic cortex and promoted anti-inflammatory skewed microglia that favored recovery [186]. However, understanding the role of PI3K-AKT signaling—and how modulating the anti-inflammatory response in microglia is shaped by extrinsic stimuli—still requires more research.

Taken together, promising research demonstrates that the PI3K-AKT pathway can be manipulated to exert neuroprotection within various disease contexts. More specifically, the pharmacological manipulation of the PI3K-AKT signaling axis can yield neuroprotective effects, further suggesting that this pathway may be a viable target for attenuating chronic neuroinflammation.

## Conclusions

The presence of chronically activated microglia within the brain is a major contributor to sustained neuroinflammation, which can amplify pathology and thereby exacerbate disease development in a range of contexts. To date, while the signaling cascades involved in derailing the microglial response are not fully defined, the PI3K-AKT signaling pathway is increasingly recognized as a key contributor to microglial activation and functioning; and thus, maladaptation of this pathway is highly implicated in this pathogenic response. Although the importance of PI3K-AKT signaling is well established in the systemic immune system, our current understanding of PI3K-AKT signaling in the neuroimmunological context remains incomplete.

This review has provided an up-to-date overview of the role of PI3K-AKT signaling in regulating microglial responses. The PI3K-AKT pathway appears to regulate both the pro- and anti-inflammatory response, with the current paradigm indicating that it serves a prominent role in initiating the production of pro-inflammatory mediators in microglia after stimulation. It then follows that, abnormal PI3K-AKT signaling in the brain can potentially derail the microglial response, promoting neurotoxicity and persistent neuroinflammation. Aging has been shown to alter PI3K-AKT signaling and dysregulation of this pathway in the brain is considered a risk factor for neurodegenerative diseases including Alzheimer’s disease and Parkinson’s disease. Whether other biological factors, such as sex—which has previously been shown to alter microglial maturation, activity and responses—can influence PI3K-AKT signaling, remains unclear [187].

Promising pharmacological studies targeting different components of the PI3K-AKT pathway suggest that this highly complex signaling cascade can be manipulated to skew the microglial response towards neuroprotection. However, further investigation into this pathway in the context of chronic neuroinflammation is needed to reveal its true potential as a viable therapeutic target for alleviating or preventing the onset of persistent neuroinflammation in injury, aging, and disease states while leaving the systemic immune system intact.

## Data Availability

Not applicable.

## Abbreviations

AKT: Protein kinase B
CNS: Central nervous system
CSF: Colony stimulating factor
CX3CR1: CX3C chemokine receptor 1
IL: Interleukin
LPS: Lipopolysaccharide
NF-κB: Nuclear factor kappa-light-chain-enhancer of activated B cells
NO: Nitric oxide
iNOS: Inducible nitric oxide synthase
PI3K: Phosphoinositide 3-kinase
PI(3,4)P_2_: Phosphatidylinositol 3,4-biphosphate
PI(4,5)P_2_: Phosphatidylinositol 4,5-biphosphate
PI(3,4,5)P_3_: Phosphatidylinositol 3,4,5-triphosphate
PTEN: Phosphatase and tensin homolog
SHIP-1: Src homology 2 domain-containing inositol polyphosphate 5-phosphatase 1
TBI: Traumatic brain injury
TLR: Toll-like receptor
TNF: Tumor necrosis factor

## References

[1] Janssen WJ, Henson PM (2012). Cellular regulation of the inflammatory response. Toxicol Pathol.

[2] Yang QQ, Zhou JW (2019). Neuroinflammation in the central nervous system: symphony of glial cells. Glia.

[3] Macháček T, Panská L, Dvořáková H, Horák P (2016). Nitric oxide and cytokine production by glial cells exposed in vitro to neuropathogenic schistosome *Trichobilharzia regenti*. Parasit Vectors.

[4] Lively S, Schlichter LC (2018). Microglia responses to pro-inflammatory stimuli (LPS, IFNγ+TNFα) and reprogramming by resolving cytokines (IL-4, IL-10). Front Cell Neurosci.

[5] von Bartheld CS, Bahney J, Herculano-Houzel S (2016). The search for true numbers of neurons and glial cells in the human brain: a review of 150 years of cell counting. J Comp Neurol.

[6] Suescun J, Chandra S, Schiess MC, Actor JK, Smith KC (2019). Chapter 13—the role of neuroinflammation in neurodegenerative disorders. Translational inflammation.

[7] Yuan N, Chen Y, Xia Y, Dai J, Liu C (2019). Inflammation-related biomarkers in major psychiatric disorders: a cross-disorder assessment of reproducibility and specificity in 43 meta-analyses. Transl Psychiatry.

[8] Schimmel SJ, Acosta S, Lozano D (2017). Neuroinflammation in traumatic brain injury: a chronic response to an acute injury. Brain Circ.

[9] Xiong Y, Mahmood A, Chopp M (2018). Current understanding of neuroinflammation after traumatic brain injury and cell-based therapeutic opportunities. Chin J Traumatol.

[10] Kumar RG, Boles JA, Wagner AK (2015). Chronic inflammation after severe traumatic brain injury: characterization and associations with outcome at 6 and 12 months postinjury. J Head Trauma Rehabil.

[11] Loane DJ, Kumar A, Stoica BA, Cabatbat R, Faden AI (2014). Progressive neurodegeneration after experimental brain trauma: association with chronic microglial activation. J Neuropathol Exp Neurol.

[12] Prinz M, Jung S, Priller J (2019). Microglia biology: one century of evolving concepts. Cell.

[13] Tay TL, Béchade C, D'Andrea I, St-Pierre MK, Henry MS, Roumier A (2017). Microglia gone rogue: impacts on psychiatric disorders across the lifespan. Front Mol Neurosci.

[14] Liddelow SA, Guttenplan KA, Clarke LE, Bennett FC, Bohlen CJ, Schirmer L (2017). Neurotoxic reactive astrocytes are induced by activated microglia. Nature.

[15] Liddelow SA, Barres BA (2017). Reactive astrocytes: production, function, and therapeutic potential. Immunity.

[16] Koenigsknecht-Talboo J, Meyer-Luehmann M, Parsadanian M, Garcia-Alloza M, Finn MB, Hyman BT (2008). Rapid microglial response around amyloid pathology after systemic anti-Abeta antibody administration in PDAPP mice. J Neurosci.

[17] Katso R, Okkenhaug K, Ahmadi K, White S, Timms J, Waterfield MD (2001). Cellular function of phosphoinositide 3-kinases: implications for development, immunity, homeostasis, and cancer. Annu Rev Cell Dev Biol.

[18] Engelman JA, Luo J, Cantley LC (2006). The evolution of phosphatidylinositol 3-kinases as regulators of growth and metabolism. Nat Rev Genet.

[19] Hawkins PT, Stephens LR (2015). PI3K signalling in inflammation. Biochim Biophys Acta.

[20] Weichhart T, Säemann MD. The PI3K/Akt/mTOR pathway in innate immune cells: emerging therapeutic applications. Ann Rheum Dis. 2008;67(Suppl 3):iii70–4. 10.1136/ard.2008.098459 19022819

[21] Xu F, Na L, Li Y, Chen L. Roles of the PI3K/AKT/mTOR signalling pathways in neurodegenerative diseases and tumours. Cell Biosci. 2020;10:54. 10.1186/s13578-020-00416-0 PMC7110906 32266056

[22] Amici SA, Dong J, Guerau-de-Arellano M (2017). Molecular mechanisms modulating the phenotype of macrophages and microglia. Front Immunol.

[23] Lawson LJ, Perry VH, Dri P, Gordon S (1990). Heterogeneity in the distribution and morphology of microglia in the normal adult mouse brain. Neuroscience.

[24] Tan Y-L, Yuan Y, Tian L (2020). Microglial regional heterogeneity and its role in the brain. Mol Psychiatry.

[25] Masuda T, Sankowski R, Staszewski O, Böttcher C, Amann L, Sagar, et al. Spatial and temporal heterogeneity of mouse and human microglia at single-cell resolution. Nature. 2019;566(7744):388–92. 10.1038/s41586-019-0924-x 30760929

[26] Li Q, Cheng Z, Zhou L, Darmanis S, Neff NF, Okamoto J (2019). Developmental heterogeneity of microglia and brain myeloid cells revealed by deep single-cell RNA sequencing. Neuron.

[27] De Biase LM, Schuebel KE, Fusfeld ZH, Jair K, Hawes IA, Cimbro R (2017). Local cues establish and maintain region-specific phenotypes of basal ganglia microglia. Neuron.

[28] Hammond TR, Dufort C, Dissing-Olesen L, Giera S, Young A, Wysoker A (2019). Single-cell RNA sequencing of microglia throughout the mouse lifespan and in the injured brain reveals complex cell-state changes. Immunity.

[29] Nimmerjahn A, Kirchhoff F, Helmchen F (2005). Resting microglial cells are highly dynamic surveillants of brain parenchyma in vivo. Science.

[30] de Fernández-Arjona MM, Grondona JM, Granados-Durán P, Fernández-Llebrez P, López-Ávalos MD (2017). Microglia morphological categorization in a rat model of neuroinflammation by hierarchical cluster and principal components analysis. Front Cell Neurosci.

[31] Keren-Shaul H, Spinrad A, Weiner A, Matcovitch-Natan O, Dvir-Szternfeld R, Ulland TK (2017). A unique microglia type associated with restricting development of Alzheimer's disease. Cell.

[32] Tay TL, Sagar, Dautzenberg J, Grün D, Prinz M. Unique microglia recovery population revealed by single-cell RNAseq following neurodegeneration. Acta Neuropathol Commun. 2018;6(1):87. 10.1186/s40478-018-0584-3 PMC6123921 30185219

[33] Pozzo ED, Tremolanti C, Costa B, Giacomelli C, Milenkovic VM, Bader S (2019). Microglial pro-inflammatory and anti-inflammatory phenotypes are modulated by translocator protein activation. Int J Mol Sci.

[34] Palin K, Cunningham C, Forse P, Perry VH, Platt N (2008). Systemic inflammation switches the inflammatory cytokine profile in CNS Wallerian degeneration. Neurobiol Dis.

[35] Morganti JM, Riparip LK, Rosi S. Call Off the Dog(ma): M1/M2 polarization is concurrent following traumatic brain injury. PLoS ONE. 2016;11(1):e0148001. 10.1371/journal.pone.0148001 PMC4726527 26808663

[36] Kim CC, Nakamura MC, Hsieh CL (2016). Brain trauma elicits non-canonical macrophage activation states. J Neuroinflamm.

[37] Orecchioni M, Ghosheh Y, Pramod AB, Ley K. Macrophage polarization: different gene signatures in M1(LPS+) vs. classically and M2(LPS-) vs. alternatively activated macrophages. Front Immunol. 2019;10:1084. 10.3389/fimmu.2019.01084 PMC6543837 31178859

[38] Bal-Price A, Brown GC (2001). Inflammatory neurodegeneration mediated by nitric oxide from activated glia-inhibiting neuronal respiration, causing glutamate release and excitotoxicity. J Neurosci.

[39] Ye L, Huang Y, Zhao L, Li Y, Sun L, Zhou Y (2013). IL-1β and TNF-α induce neurotoxicity through glutamate production: a potential role for neuronal glutaminase. J Neurochem.

[40] Stojakovic A, Paz-Filho G, Arcos-Burgos M, Licinio J, Wong M-L, Mastronardi CA (2017). Role of the IL-1 pathway in dopaminergic neurodegeneration and decreased voluntary movement. Mol Neurobiol.

[41] Weekman EM, Sudduth TL, Abner EL, Popa GJ, Mendenhall MD, Brothers HM (2014). Transition from an M1 to a mixed neuroinflammatory phenotype increases amyloid deposition in APP/PS1 transgenic mice. J Neuroinflamm.

[42] Che Y, Hou L, Sun F, Zhang C, Liu X, Piao F (2018). Taurine protects dopaminergic neurons in a mouse Parkinson's disease model through inhibition of microglial M1 polarization. Cell Death Dis.

[43] Valera E, Mante M, Anderson S, Rockenstein E, Masliah E (2015). Lenalidomide reduces microglial activation and behavioral deficits in a transgenic model of Parkinson’s disease. J Neuroinflamm.

[44] Sapp E, Kegel KB, Aronin N, Hashikawa T, Uchiyama Y, Tohyama K (2001). Early and progressive accumulation of reactive microglia in the Huntington disease brain. J Neuropathol Exp Neurol.

[45] Olesen MN, Wuolikainen A, Nilsson AC, Wirenfeldt M, Forsberg K, Madsen JS, et al. Inflammatory profiles relate to survival in subtypes of amyotrophic lateral sclerosis. Neurol Neuroimmunol Neuroinflamm. 2020;7(3):e697. 10.1212/NXI.0000000000000697 PMC7136052 32123048

[46] Ramlackhansingh AF, Brooks DJ, Greenwood RJ, Bose SK, Turkheimer FE, Kinnunen KM (2011). Inflammation after trauma: microglial activation and traumatic brain injury. Ann Neurol.

[47] Streit WJ, Mrak RE, Griffin WST. Microglia and neuroinflammation: a pathological perspective. J Neuroinflamm. 2004;1(1):14. 10.1186/1742-2094-1-14 PMC509427 15285801

[48] Crotti A, Glass CK (2015). The choreography of neuroinflammation in Huntington's disease. Trends Immunol.

[49] Benatti C, Blom JMC, Rigillo G, Alboni S, Zizzi F, Torta R (2016). Disease-induced neuroinflammation and depression. CNS Neurol Disord Drug Targets.

[50] Wagner AK, Amin KB, Niyonkuru C, Postal BA, McCullough EH, Ozawa H (2011). CSF Bcl-2 and cytochrome C temporal profiles in outcome prediction for adults with severe TBI. J Cereb Blood Flow Metab.

[51] Grossetete M, Phelps J, Arko L, Yonas H, Rosenberg GA (2009). Elevation of matrix metalloproteinases 3 and 9 in cerebrospinal fluid and blood in patients with severe traumatic brain injury. Neurosurgery.

[52] Hergenroeder GW, Moore AN, McCoy JP, Samsel L, Ward NH, Clifton GL (2010). Serum IL-6: a candidate biomarker for intracranial pressure elevation following isolated traumatic brain injury. J Neuroinflamm.

[53] Xiao J, Yao R, Xu B, Wen H, Zhong J, Li D (2020). Inhibition of PDE4 attenuates TNF-α-triggered cell death through suppressing NF-κB and JNK activation in HT-22 neuronal cells. Cell Mol Neurobiol.

[54] Brown AD, Fogarty MJ, Mantilla CB, Sieck GC (2019). The effect of TNF-α on mitochondrial morphology in model (NSC-34) motor neurons. FASEB J.

[55] Sama DM, Norris CM (2013). Calcium dysregulation and neuroinflammation: discrete and integrated mechanisms for age-related synaptic dysfunction. Ageing Res Rev.

[56] Walker KA (2018). Inflammation and neurodegeneration: chronicity matters. Aging.

[57] Villegas-Llerena C, Phillips A, Garcia-Reitboeck P, Hardy J, Pocock JM (2016). Microglial genes regulating neuroinflammation in the progression of Alzheimer's disease. Curr Opin Neurobiol.

[58] Rai SN, Dilnashin H, Birla H, Singh SS, Zahra W, Rathore AS (2019). The role of PI3K/Akt and ERK in neurodegenerative disorders. Neurotox Res.

[59] Heras-Sandoval D, Pérez-Rojas JM, Hernández-Damián J, Pedraza-Chaverri J (2014). The role of PI3K/AKT/mTOR pathway in the modulation of autophagy and the clearance of protein aggregates in neurodegeneration. Cell Signal.

[60] Fruman DA, Chiu H, Hopkins BD, Bagrodia S, Cantley LC, Abraham RT (2017). The PI3K pathway in human disease. Cell.

[61] Hemmings BA, Restuccia DF. PI3K-PKB/Akt pathway. Cold Spring Harb Perspect Biol. 2012;4(9):a011189. 10.1101/cshperspect.a011189 PMC3428770 22952397

[62] Cianciulli A, Porro C, Calvello R, Trotta T, Lofrumento DD, Panaro MA (2020). Microglia mediated neuroinflammation: focus on PI3K modulation. Biomolecules.

[63] Xiao L, Gong LL, Yuan D, Deng M, Zeng XM, Chen LL (2010). Protein phosphatase-1 regulates Akt1 signal transduction pathway to control gene expression, cell survival and differentiation. Cell Death Differ.

[64] Scheid MP, Woodgett JR (2001). PKB/AKT: functional insights from genetic models. Nat Rev Mol Cell Biol.

[65] Zhou H, Li XM, Meinkoth J, Pittman RN (2000). Akt regulates cell survival and apoptosis at a postmitochondrial level. J Cell Biol.

[66] Chang F, Lee JT, Navolanic PM, Steelman LS, Shelton JG, Blalock WL (2003). Involvement of PI3K/Akt pathway in cell cycle progression, apoptosis, and neoplastic transformation: a target for cancer chemotherapy. Leukemia.

[67] Xie S, Chen M, Yan B, He X, Chen X, Li D. Identification of a role for the PI3K/AKT/mTOR signaling pathway in innate immune cells. PLoS ONE. 2014;9(4):e94496. 10.1371/journal.pone.0094496 PMC3981814 24718556

[68] Koyasu S (2003). The role of PI3K in immune cells. Nat Immunol.

[69] Jiang N, Dai Q, Su X, Fu J, Feng X, Peng J (2020). Role of PI3K/AKT pathway in cancer: the framework of malignant behavior. Mol Biol Rep.

[70] Suzuki Y, Shirai K, Oka K, Mobaraki A, Yoshida Y, Noda SE (2010). Higher pAkt expression predicts a significant worse prognosis in glioblastomas. J Radiat Res.

[71] Kim WK, Hwang SY, Oh ES, Piao HZ, Kim KW, Han IO. TGF-beta1 represses activation and resultant death of microglia via inhibition of phosphatidylinositol 3-kinase activity. J Immunol (Baltimore, Md: 1950). 2004;172(11):7015–23. 10.4049/jimmunol.172.11.7015 15153523

[72] Ito S, Sawada M, Haneda M, Ishida Y, Isobe K-i. Amyloid-β peptides induce several chemokine mRNA expressions in the primary microglia and Ra2 cell line via the PI3K/Akt and/or ERK pathway. Neurosc Res. 2006;56(3):294–9. 10.1016/j.neures.2006.07.009 16978723

[73] Saponaro C, Cianciulli A, Calvello R, Dragone T, Iacobazzi F, Panaro MA (2012). The PI3K/Akt pathway is required for LPS activation of microglial cells. Immunopharmacol Immunotoxicol.

[74] Tarassishin L, Suh HS, Lee SC (2011). Interferon regulatory factor 3 plays an anti-inflammatory role in microglia by activating the PI3K/Akt pathway. J Neuroinflamm.

[75] Hoogland ICM, Westhoff D, Engelen-Lee J-Y, Melief J, Valls Serón M, Houben-Weerts JHMP (2018). Microglial activation after systemic stimulation with lipopolysaccharide and *Escherichia coli*. Front Cell Neurosci.

[76] Willis EF, MacDonald KPA, Nguyen QH, Garrido AL, Gillespie ER, Harley SBR (2020). Repopulating microglia promote brain repair in an IL-6-dependent manner. Cell.

[77] Acaz-Fonseca E, Ortiz-Rodriguez A, Azcoitia I, Garcia-Segura LM, Arevalo M-A (2019). Notch signaling in astrocytes mediates their morphological response to an inflammatory challenge. Cell Death Discov.

[78] Bhat SA, Henry RJ, Blanchard AC, Stoica BA, Loane DJ, Faden AI. Enhanced Akt/GSK-3β/CREB signaling mediates the anti-inflammatory actions of mGluR5 positive allosteric modulators in microglia and following traumatic brain injury in male mice. J Neurochem. 2021;156(2):225–248. 10.1111/jnc.14954 PMC7386074 31926033

[79] Lissner MM, Thomas BJ, Wee K, Tong A-J, Kollmann TR, Smale ST. Age-related gene expression differences in monocytes from human neonates, young adults, and older adults. PLoS ONE. 2015;10(7):e0132061. 10.1371/journal.pone.0132061 PMC4493075 26147648

[80] Krow-Lucal ER, Kim CC, Burt TD, McCune JM (2014). Distinct functional programming of human fetal and adult monocytes. Blood.

[81] Njie EG, Boelen E, Stassen FR, Steinbusch HWM, Borchelt DR, Streit WJ. Ex vivo cultures of microglia from young and aged rodent brain reveal age-related changes in microglial function. Neurobiol Aging. 2012;33(1):195.e1–12. 10.1016/j.neurobiolaging.2010.05.008 PMC4162517 20580465

[82] Orellana AMM, Vasconcelos AR, Leite JA, de Sá LL, Andreotti DZ, Munhoz CD (2015). Age-related neuroinflammation and changes in AKT-GSK-3β and WNT/ β-CATENIN signaling in rat hippocampus. Aging.

[83] Doens D, Fernández PL (2014). Microglia receptors and their implications in the response to amyloid β for Alzheimer’s disease pathogenesis. J Neuroinflamm.

[84] Bachiller S, Jiménez-Ferrer I, Paulus A, Yang Y, Swanberg M, Deierborg T (2018). Microglia in neurological diseases: a road map to brain-disease dependent-inflammatory response. Front Cell Neurosci.

[85] Xu S, Wang J, Jiang J, Song J, Zhu W, Zhang F (2020). TLR4 promotes microglial pyroptosis via lncRNA-F630028O10Rik by activating PI3K/AKT pathway after spinal cord injury. Cell Death Dis.

[86] Oosterhof N, Chang IJ, Karimiani EG, Kuil LE, Jensen DM, Daza R (2019). Homozygous mutations in CSF1R cause a pediatric-onset leukoencephalopathy and can result in congenital absence of microglia. Am J Hum Genet.

[87] Konno T, Tada M, Tada M, Koyama A, Nozaki H, Harigaya Y (2014). Haploinsufficiency of *CSF-1R* and clinicopathologic characterization in patients with HDLS. Neurology.

[88] Erblich B, Zhu L, Etgen AM, Dobrenis K, Pollard JW. Absence of colony stimulation factor-1 receptor results in loss of microglia, disrupted brain development and olfactory deficits. PLoS ONE. 2011;6(10):e26317. 10.1371/journal.pone.0026317 PMC3203114 22046273

[89] Hagan N, Kane JL, Grover D, Woodworth L, Madore C, Saleh J (2020). CSF1R signaling is a regulator of pathogenesis in progressive MS. Cell Death Dis.

[90] Pons V, Lévesque P, Plante M-M, Rivest S. Conditional genetic deletion of CSF1 receptor in microglia ameliorates the physiopathology of Alzheimer's disease. Alzheimer's Res Ther. 2021;13(1):8. 10.1186/s13195-020-00747-7 PMC7783991 33402196

[91] Cardona AE, Pioro EP, Sasse ME, Kostenko V, Cardona SM, Dijkstra IM (2006). Control of microglial neurotoxicity by the fractalkine receptor. Nat Neurosci.

[92] Rottlaender A, Villwock H, Addicks K, Kuerten S (2011). Neuroprotective role of fibroblast growth factor-2 in experimental autoimmune encephalomyelitis. Immunology.

[93] Rossi C, Cusimano M, Zambito M, Finardi A, Capotondo A, Garcia-Manteiga JM (2018). Interleukin 4 modulates microglia homeostasis and attenuates the early slowly progressive phase of amyotrophic lateral sclerosis. Cell Death Dis.

[94] Gadani SP, Cronk JC, Norris GT, Kipnis J. IL-4 in the brain: a cytokine to remember. J Immunol (Baltimore, Md: 1950). 2012;189(9):4213–9. 10.4049/jimmunol.1202246 PMC3481177 23087426

[95] Gabbouj S, Ryhänen S, Marttinen M, Wittrahm R, Takalo M, Kemppainen S (2019). Altered insulin signaling in Alzheimer’s disease brain—special emphasis on PI3K-Akt pathway. Front Neurosci.

[96] Griffith CM, Eid T, Rose GM, Patrylo PR (2018). Evidence for altered insulin receptor signaling in Alzheimer's disease. Neuropharmacology.

[97] Go M, Kou J, Lim JE, Yang J, Fukuchi KI (2016). Microglial response to LPS increases in wild-type mice during aging but diminishes in an Alzheimer's mouse model: implication of TLR4 signaling in disease progression. Biochem Biophys Res Commun.

[98] Lee JY, Lee JD, Phipps S, Noakes PG, Woodruff TM. Absence of toll-like receptor 4 (TLR4) extends survival in the hSOD1 G93A mouse model of amyotrophic lateral sclerosis. J neuroinflamm. 2015;12:90. 10.1186/s12974-015-0310-z PMC4431460 25962427

[99] Shin W-H, Jeon M-T, Leem E, Won S-Y, Jeong KH, Park S-J (2015). Induction of microglial toll-like receptor 4 by prothrombin kringle-2: a potential pathogenic mechanism in Parkinson’s disease. Sci Rep.

[100] Dardiotis E, Siokas V, Pantazi E, Dardioti M, Rikos D, Xiromerisiou G, et al. A novel mutation in TREM2 gene causing Nasu–Hakola disease and review of the literature. Neurobiol Aging. 2017;53:194.e13–22. 10.1016/j.neurobiolaging.2017.01.015 28214109

[101] Jonsson T, Stefansson H, Steinberg S, Jonsdottir I, Jonsson PV, Snaedal J (2013). Variant of TREM2 associated with the risk of Alzheimer's disease. N Engl J Med.

[102] Guerreiro R, Wojtas A, Bras J, Carrasquillo M, Rogaeva E, Majounie E (2013). TREM2 variants in Alzheimer's disease. N Engl J Med.

[103] Jiang T, Yu J-T, Zhang Y-D, Tan L. A rare coding variant in *TREM2* increases risk for Alzheimer’s disease in Han Chinese (P5.164). Neurology. 2016;86(16 Supplement):P5.164. 10.1016/j.neurobiolaging.2016.02.023 27067662

[104] Lin W, Xu D, Austin CD, Caplazi P, Senger K, Sun Y (2019). Function of CSF1 and IL34 in macrophage homeostasis, inflammation, and cancer. Front Immunol.

[105] Rosin JM, Vora SR, Kurrasch DM (2018). Depletion of embryonic microglia using the CSF1R inhibitor PLX5622 has adverse sex-specific effects on mice, including accelerated weight gain, hyperactivity and anxiolytic-like behaviour. Brain Behav Immun.

[106] Spangenberg E, Severson PL, Hohsfield LA, Crapser J, Zhang J, Burton EA (2019). Sustained microglial depletion with CSF1R inhibitor impairs parenchymal plaque development in an Alzheimer's disease model. Nat Commun.

[107] Boulakirba S, Pfeifer A, Mhaidly R, Obba S, Goulard M, Schmitt T (2018). IL-34 and CSF-1 display an equivalent macrophage differentiation ability but a different polarization potential. Sci Rep.

[108] Li W, Stanley ER (1991). Role of dimerization and modification of the CSF-1 receptor in its activation and internalization during the CSF-1 response. EMBO J.

[109] Sampaio NG, Yu W, Cox D, Wyckoff J, Condeelis J, Stanley ER (2011). Phosphorylation of CSF-1R Y721 mediates its association with PI3K to regulate macrophage motility and enhancement of tumor cell invasion. J Cell Sci.

[110] Ulland TK, Wang Y, Colonna M (2015). Regulation of microglial survival and proliferation in health and diseases. Semin Immunol.

[111] Pepe G, De Maglie M, Minoli L, Villa A, Maggi A, Vegeto E (2017). Selective proliferative response of microglia to alternative polarization signals. J Neuroinflamm.

[112] Olmos-Alonso A, Schetters ST, Sri S, Askew K, Mancuso R, Vargas-Caballero M (2016). Pharmacological targeting of CSF1R inhibits microglial proliferation and prevents the progression of Alzheimer's-like pathology. Brain.

[113] Neal ML, Fleming SM, Budge KM, Boyle AM, Kim C, Alam G (2020). Pharmacological inhibition of CSF1R by GW2580 reduces microglial proliferation and is protective against neuroinflammation and dopaminergic neurodegeneration. FASEB J.

[114] Konishi H, Kiyama H (2018). Microglial TREM2/DAP12 signaling: a double-edged sword in neural diseases. Front Cell Neurosci.

[115] Horti AG, Naik R, Foss CA, Minn I, Misheneva V, Du Y (2019). PET imaging of microglia by targeting macrophage colony-stimulating factor 1 receptor (CSF1R). Proc Natl Acad Sci USA.

[116] Blevins G, Fedoroff S (1995). Microglia in colony-stimulating factor 1-deficient op/op mice. J Neurosci Res.

[117] Ginhoux F, Greter M, Leboeuf M, Nandi S, See P, Gokhan S (2010). Fate mapping analysis reveals that adult microglia derive from primitive macrophages. Science (New York, NY).

[118] Lin H, Lee E, Hestir K, Leo C, Huang M, Bosch E (2008). Discovery of a cytokine and its receptor by functional screening of the extracellular proteome. Science.

[119] van Noort JM, Bsibsi M, Verhaagen J, Hol EM, Huitenga I, Wijnholds J, Bergen AB, Boer GJ (2009). Toll-like receptors in the CNS: implications for neurodegeneration and repair. Progress in brain research.

[120] Fiebich BL, Batista CRA, Saliba SW, Yousif NM, de Oliveira ACP. Role of microglia TLRs in neurodegeneration. Front Cell Neurosci. 2018;12:329. 10.3389/fncel.2018.00329 PMC6176466 30333729

[121] Song M, Jin J, Lim J-E, Kou J, Pattanayak A, Rehman JA (2011). TLR4 mutation reduces microglial activation, increases Aβ deposits and exacerbates cognitive deficits in a mouse model of Alzheimer's disease. J Neuroinflamm.

[122] Cui W, Sun C, Ma Y, Wang S, Wang X, Zhang Y (2020). Inhibition of TLR4 induces M2 microglial polarization and provides neuroprotection via the NLRP3 inflammasome in Alzheimer’s disease. Front Neurosci.

[123] Jack CS, Arbour N, Manusow J, Montgrain V, Blain M, McCrea E (2005). TLR signaling tailors innate immune responses in human microglia and astrocytes. J Immunol.

[124] Laird MHW, Rhee SH, Perkins DJ, Medvedev AE, Piao W, Fenton MJ (2009). TLR4/MyD88/PI3K interactions regulate TLR4 signaling. J Leukoc Biol.

[125] Shao QH, Yan WF, Zhang Z, Ma KL, Peng SY, Cao YL (2019). Nurr1: a vital participant in the TLR4-NF-κB signal pathway stimulated by α-synuclein in BV-2 cells. Neuropharmacology.

[126] Ryu K-Y, Lee H-j, Woo H, Kang R-J, Han K-M, Park H, et al. Dasatinib regulates LPS-induced microglial and astrocytic neuroinflammatory responses by inhibiting AKT/STAT3 signaling. J Neuroinflamm. 2019;16(1):190. 10.1186/s12974-019-1561-x PMC6815018 31655606

[127] Qin Y, Liu Y, Hao W, Decker Y, Tomic I, Menger MD, et al. Stimulation of TLR4 attenuates Alzheimer's disease-related symptoms and pathology in tau-transgenic mice. J Immunol (Baltimore, Md: 1950). 2016;197(8):3281–92. 10.4049/jimmunol.1600873 27605009

[128] Stefanova N, Fellner L, Reindl M, Masliah E, Poewe W, Wenning GK (2011). Toll-like receptor 4 promotes α-synuclein clearance and survival of nigral dopaminergic neurons. Am J Pathol.

[129] Calvo-Rodriguez M, García-Rodríguez C, Villalobos C, Núñez L (2020). Role of toll like receptor 4 in Alzheimer’s disease. Front Immunol.

[130] Campolo M, Paterniti I, Siracusa R, Filippone A, Esposito E, Cuzzocrea S (2019). TLR4 absence reduces neuroinflammation and inflammasome activation in Parkinson's diseases in vivo model. Brain Behav Immun.

[131] Hughes CD, Choi ML, Ryten M, Hopkins L, Drews A, Botía JA (2019). Picomolar concentrations of oligomeric alpha-synuclein sensitizes TLR4 to play an initiating role in Parkinson’s disease pathogenesis. Acta Neuropathol.

[132] Turnbull IR, Gilfillan S, Cella M, Aoshi T, Miller M, Piccio L (2006). Cutting edge: TREM-2 attenuates macrophage activation. J Immunol.

[133] Kober DL, Brett TJ (2017). TREM2-ligand interactions in health and disease. J Mol Biol.

[134] Peng Q, Malhotra S, Humphrey MB. Association of TREM2-DAP12 with DAP10 is required for the regulation of PI3K in macrophages (98.18). J Immunol. 2010;184(1 Supplement):98.18.

[135] Ulland TK, Song WM, Huang SC-C, Ulrich JD, Sergushichev A, Beatty WL, et al. TREM2 maintains microglial metabolic fitness in Alzheimer’s disease. Cell. 2017;170(4):649–63.e13. 10.1016/j.cell.2017.07.023 PMC5573224 28802038

[136] Li C, Zhao B, Lin C, Gong Z, An X (2019). TREM2 inhibits inflammatory responses in mouse microglia by suppressing the PI3K/NF-κB signaling. Cell Biol Int.

[137] Kim S-M, Mun B-R, Lee S-J, Joh Y, Lee H-Y, Ji K-Y (2017). TREM2 promotes Aβ phagocytosis by upregulating C/EBPα-dependent CD36 expression in microglia. Sci Rep.

[138] Zheng H, Jia L, Liu C-C, Rong Z, Zhong L, Yang L (2017). TREM2 promotes microglial survival by activating Wnt/β-catenin pathway. J Neurosci.

[139] Lessard CB, Malnik SL, Zhou Y, Ladd TB, Cruz PE, Ran Y (2018). High-affinity interactions and signal transduction between Aβ oligomers and TREM2. EMBO Mol Med.

[140] Xing J, Titus AR, Humphrey MB (2015). The TREM2-DAP12 signaling pathway in Nasu–Hakola disease: a molecular genetics perspective. Res Rep Biochem.

[141] Zhao Y, Wu X, Li X, Jiang LL, Gui X, Liu Y (2018). TREM2 is a receptor for β-amyloid that mediates microglial function. Neuron.

[142] Gratuze M, Chen Y, Parhizkar S, Jain N, Strickland MR, Serrano JR (2021). Activated microglia mitigate Aβ-associated tau seeding and spreading. J Exp Med.

[143] Wang W-Y, Tan M-S, Yu J-T, Tan L (2015). Role of pro-inflammatory cytokines released from microglia in Alzheimer's disease. Ann Transl Med.

[144] Harrison JK, Jiang Y, Chen S, Xia Y, Maciejewski D, McNamara RK (1998). Role for neuronally derived fractalkine in mediating interactions between neurons and CX3CR1-expressing microglia. Proc Natl Acad Sci USA.

[145] Wolf Y, Yona S, Kim K-W, Jung S (2013). Microglia, seen from the CX3CR1 angle. Front Cell Neurosci.

[146] Lyons A, Lynch AM, Downer EJ, Hanley R, O’Sullivan JB, Smith A (2009). Fractalkine-induced activation of the phosphatidylinositol-3 kinase pathway attentuates microglial activation in vivo and in vitro. J Neurochem.

[147] Chidambaram H, Das R, Chinnathambi S (2020). Interaction of Tau with the chemokine receptor, CX3CR1 and its effect on microglial activation, migration and proliferation. Cell Biosci.

[148] Febinger HY, Thomasy HE, Pavlova MN, Ringgold KM, Barf PR, George AM (2015). Time-dependent effects of CX3CR1 in a mouse model of mild traumatic brain injury. J Neuroinflamm.

[149] Pabon MM, Bachstetter AD, Hudson CE, Gemma C, Bickford PC (2011). CX3CL1 reduces neurotoxicity and microglial activation in a rat model of Parkinson's disease. J Neuroinflamm.

[150] Nash KR, Lee DC, Hunt JB, Morganti JM, Selenica ML, Moran P (2013). Fractalkine overexpression suppresses tau pathology in a mouse model of tauopathy. Neurobiol Aging.

[151] Efthymiou AG, Goate AM. Late onset Alzheimer's disease genetics implicates microglial pathways in disease risk. Mol Neurodegener. 2017;12(1):43. 10.1186/s13024-017-0184-x PMC5446752 28549481

[152] Yao X, Risacher SL, Nho K, Saykin AJ, Wang Z, Shen L (2019). Targeted genetic analysis of cerebral blood flow imaging phenotypes implicates the INPP5D gene. Neurobiol Aging.

[153] Farfel JM, Yu L, Buchman AS, Schneider JA, De Jager PL, Bennett DA (2016). Relation of genomic variants for Alzheimer disease dementia to common neuropathologies. Neurology.

[154] Yoshino Y, Yamazaki K, Ozaki Y, Sao T, Yoshida T, Mori T (2017). INPP5D mRNA expression and cognitive decline in Japanese Alzheimer's disease subjects. J Alzheimer's Dis.

[155] Tsai AP, Lin PB-C, Dong C, Moutinho M, Casali BT, Liu Y, et al. INPP5D expression is associated with risk for Alzheimer's disease and induced by plaque-associated microglia. Neurobiol Dis. 2021;153:105303. 10.1016/j.nbd.2021.105303 PMC8082515 33631273

[156] Träger U, Andre R, Lahiri N, Magnusson-Lind A, Weiss A, Grueninger S (2014). HTT-lowering reverses Huntington’s disease immune dysfunction caused by NFκB pathway dysregulation. Brain.

[157] Colin E, Régulier E, Perrin V, Dürr A, Brice A, Aebischer P (2005). Akt is altered in an animal model of Huntington's disease and in patients. Eur J Neurosci.

[158] Venero JL, Burguillos MA, Brundin P, Joseph B (2011). The executioners sing a new song: killer caspases activate microglia. Cell Death Differ.

[159] Yang H-M, Yang S, Huang S-S, Tang B-S, Guo J-F. Microglial activation in the pathogenesis of Huntington's disease. Front Aging neurosci. 2017;9:193. 10.3389/fnagi.2017.00193 PMC5474461 28674491

[160] Jha SK, Jha NK, Kar R, Ambasta RK, Kumar P (2015). p38 MAPK and PI3K/AKT signalling cascades in Parkinson's disease. Int J Mol Cell Med.

[161] Valente EM, Abou-Sleiman PM, Caputo V, Muqit MM, Harvey K, Gispert S (2004). Hereditary early-onset Parkinson's disease caused by mutations in PINK1. Science.

[162] Kim J, Byun J-W, Choi I, Kim B, Jeong H-K, Jou I (2013). PINK1 deficiency enhances inflammatory cytokine release from acutely prepared brain slices. Exp Neurobiol.

[163] Sun L, Shen R, Agnihotri SK, Chen Y, Huang Z, Büeler H (2018). Lack of PINK1 alters glia innate immune responses and enhances inflammation-induced, nitric oxide-mediated neuron death. Sci Rep.

[164] Jing H, Zhu J-X, Wang H-F, Zhang W, Zheng Z-J, Kong L-L (2016). INPP5D rs35349669 polymorphism with late-onset Alzheimer's disease: a replication study and meta-analysis. Oncotarget.

[165] Gjoneska E, Pfenning AR, Mathys H, Quon G, Kundaje A, Tsai L-H (2015). Conserved epigenomic signals in mice and humans reveal immune basis of Alzheimer's disease. Nature.

[166] Maxwell MJ, Duan M, Armes JE, Anderson GP, Tarlinton DM, Hibbs ML (2011). Genetic segregation of inflammatory lung disease and autoimmune disease severity in SHIP-1−/− mice. J Immunol.

[167] Hibbs ML, Raftery AL, Tsantikos E (2018). Regulation of hematopoietic cell signaling by SHIP-1 inositol phosphatase: growth factors and beyond. Growth Factors.

[168] Furlong RM, Lindsay A, Anderson KE, Hawkins PT, Sullivan AM, O'Neill C (2019). The Parkinson's disease gene PINK1 activates Akt via PINK1 kinase-dependent regulation of the phospholipid PI(3,4,5)P3. J Cell Sci.

[169] Torres-Odio S, Key J, Hoepken H-H, Canet-Pons J, Valek L, Roller B (2017). Progression of pathology in PINK1-deficient mouse brain from splicing via ubiquitination, ER stress, and mitophagy changes to neuroinflammation. J Neuroinflamm.

[170] Barodia SK, McMeekin LJ, Creed RB, Quinones EK, Cowell RM, Goldberg MS. PINK1 phosphorylates ubiquitin predominantly in astrocytes. npj Parkinson's Dis. 2019;5(1):29. 10.1038/s41531-019-0101-9 PMC6906478 31840043

[171] Wang Y, Mao X, Chen H, Feng J, Yan M, Wang Y (2019). Dexmedetomidine alleviates LPS-induced apoptosis and inflammation in macrophages by eliminating damaged mitochondria via PINK1 mediated mitophagy. Int Immunopharmacol.

[172] Culmsee C, Michels S, Scheu S, Arolt V, Dannlowski U, Alferink J (2019). Mitochondria, microglia, and the immune system—how are they linked in affective disorders?. Front Psychiatry.

[173] Humbert S, Bryson EA, Cordelières FP, Connors NC, Datta SR, Finkbeiner S (2002). The IGF-1/Akt pathway is neuroprotective in Huntington's disease and involves Huntingtin phosphorylation by Akt. Dev Cell.

[174] Venero JL, Burguillos MA, Joseph B (2013). Caspases playing in the field of neuroinflammation: old and new players. Dev Neurosci.

[175] Burguillos MA, Deierborg T, Kavanagh E, Persson A, Hajji N, Garcia-Quintanilla A (2011). Caspase signalling controls microglia activation and neurotoxicity. Nature.

[176] Erener S, Pétrilli V, Kassner I, Minotti R, Castillo R, Santoro R (2012). Inflammasome-activated caspase 7 cleaves PARP1 to enhance the expression of a subset of NF-κB target genes. Mol Cell.

[177] Dong H, Zhang X, Dai X, Lu S, Gui B, Jin W (2014). Lithium ameliorates lipopolysaccharide-induced microglial activation via inhibition of toll-like receptor 4 expression by activating the PI3K/Akt/FoxO1 pathway. J Neuroinflamm.

[178] Huang HY, Chang HF, Tsai MJ, Chen JS, Wang MJ (2016). 6-Mercaptopurine attenuates tumor necrosis factor-α production in microglia through Nur77-mediated transrepression and PI3K/Akt/mTOR signaling-mediated translational regulation. J Neuroinflamm.

[179] Jung J-S, Choi M-J, Lee YY, Moon B-I, Park J-S, Kim H-S (2017). Suppression of lipopolysaccharide-induced neuroinflammation by morin via MAPK, PI3K/Akt, and PKA/HO-1 signaling pathway modulation. J Agric Food Chem.

[180] Martínez-Mármol R, Mohannak N, Qian L, Wang T, Gormal RS, Ruitenberg MJ (2019). p110δ PI3-kinase inhibition perturbs APP and TNFα trafficking, reduces plaque burden, dampens neuroinflammation, and prevents cognitive decline in an Alzheimer's disease mouse model. J Neurosci.

[181] Wang P, He Y, Li D, Han R, Liu G, Kong D (2016). Class I PI3K inhibitor ZSTK474 mediates a shift in microglial/macrophage phenotype and inhibits inflammatory response in mice with cerebral ischemia/reperfusion injury. J Neuroinflamm.

[182] Kim BW, Koppula S, Kumar H, Park JY, Kim IW, More SV (2015). α-Asarone attenuates microglia-mediated neuroinflammation by inhibiting NF kappa B activation and mitigates MPTP-induced behavioral deficits in a mouse model of Parkinson's disease. Neuropharmacology.

[183] Wang S, Jing H, Yang H, Liu Z, Guo H, Chai L (2015). Tanshinone I selectively suppresses pro-inflammatory genes expression in activated microglia and prevents nigrostriatal dopaminergic neurodegeneration in a mouse model of Parkinson's disease. J Ethnopharmacol.

[184] Koo J-H, Jang Y-C, Hwang D-J, Um H-S, Lee N-H, Jung J-H (2017). Treadmill exercise produces neuroprotective effects in a murine model of Parkinson’s disease by regulating the TLR2/MyD88/NF-κB signaling pathway. Neuroscience.

[185] Li YH, Fu HL, Tian ML, Wang YQ, Chen W, Cai LL (2016). Neuron-derived FGF10 ameliorates cerebral ischemia injury via inhibiting NF-κB-dependent neuroinflammation and activating PI3K/Akt survival signaling pathway in mice. Sci Rep.

[186] Baba T, Kameda M, Yasuhara T, Morimoto T, Kondo A, Shingo T (2009). Electrical stimulation of the cerebral cortex exerts antiapoptotic, angiogenic, and anti-inflammatory effects in ischemic stroke rats through phosphoinositide 3-kinase/Akt signaling pathway. Stroke.

[187] Bordt EA, Ceasrine AM, Bilbo SD (2020). Microglia and sexual differentiation of the developing brain: a focus on ontogeny and intrinsic factors. Glia.

